# Human Leukocyte Antigen Class II Haplotypes Affect Clinical Characteristics and Progression of Type 1 Autoimmune Hepatitis in Japan

**DOI:** 10.1371/journal.pone.0100565

**Published:** 2014-06-23

**Authors:** Takeji Umemura, Yoshihiko Katsuyama, Kaname Yoshizawa, Takefumi Kimura, Satoru Joshita, Michiharu Komatsu, Akihiro Matsumoto, Eiji Tanaka, Masao Ota

**Affiliations:** 1 Department of Medicine, Division of Hepatology and Gastroenterology, Shinshu University School of Medicine, Matsumoto, Japan; 2 Department of Pharmacy, Shinshu University Hospital, Matsumoto, Japan; 3 Department of Legal Medicine, Shinshu University School of Medicine, Matsumoto, Japan; 4 Department of Gastroenterology, NHO Ueda Medical Center, Ueda, Japan; Centro di Riferimento Oncologico, IRCCS National Cancer Institute, Italy

## Abstract

Although we earlier demonstrated that the human leukocyte antigen (HLA) DRB1*04:05 allele was associated with susceptibility to autoimmune hepatitis (AIH) in Japan, the precise relationship of HLA haplotype and the role of amino acid alignment with disease susceptibility and progression has not been fully clarified. We reinvestigated HLA class I *A*, *B*, and *C* and HLA class II *DRB1*, *DQB1*, and *DPB1* alleles and haplotypes in a larger new cohort of 156 Japanese patients with type 1 AIH and compared them with the published data of 210 healthy subjects. The DRB1*04:05-DQB1*04:01 haplotype was significantly associated with AIH susceptibility (30% vs. 11%, *P* = 1.2×10^−10^; odds ratio [OR]  = 3.51) and correlated with elevated serum IgG (3042 vs. 2606 mg/dL, *P* = 0.041) and anti-smooth muscle antigen positivity (77% vs. 34%, *P* = 0.000006). No associations with *HLA-DPB1* alleles were found. The HLA A*24:02 and C*01:02 alleles were associated with disease susceptibility (corrected *P* = 0.0053 and 0.036, respectively), but this likely constituents of a long ranged haplotype including DRB1*04:05-DQB1*04:01 haplotype. Conversely, the DRB1*15:01-DQB1*06:02 haplotype was associated with protection from both disease onset (5% vs. 13%, *P* = 0.00057; OR = 0.38) and the development of hepatocellular carcinoma (25% vs. 5%, *P* = 0.017; OR = 6.81). The frequency of the DRB1*08:03-DQB1*06:01 haplotype was significantly higher in patients who developed hepatic failure (22% vs. 6%, *P* = 0.034; OR = 4.38). In conclusion, this study established the role of HLA haplotypes in determining AIH susceptibility and progression in the Japanese population. Additional sequencing of the entire HLA region is required to more precisely identify the genetic components of AIH.

## Introduction

Autoimmune hepatitis (AIH) is characterized by chronic inflammation of the liver, elevated transaminase levels, hypergammaglobulinemia, serum autoantibodies, histologic evidence of interface hepatitis, and a favorable response to immunosuppressive treatment.[1]–[3] Although this disease is believed to result from a combination of genetic and environmental factors, its exact etiology remains unclear. In previous studies, the HLA DRB1*04:05-DQB1*04:01 haplotype in Japanese [4], [5] and the DRB1*03:01 and/or DRB1*04:01 alleles in Caucasians [6]–[8] were identified as independent determinants of susceptibility to type 1 AIH. Czaja et al.[8] reported that the HLA DRB1*03:01 allele was associated with a poor treatment response and that DRB1*04:01 was related to a lower frequency of hepatic death or transplantation in Caucasians, but associations between HLA alleles and haplotypes and clinical manifestations were not investigated. Recent long-term follow-up studies have also shown that hepatic failure and hepatocellular carcinoma (HCC) complicating AIH are not as rare as earlier believed;[9], [10] however, the genetic predisposition to advanced liver diseases has not been addressed. Strettell et al.[11] found that the HLA-Cw*07:01 allele contributed to disease susceptibility in England, although no supporting data has been reported to date. It was recently proposed that associations with specific HLA-C and HLA-B alleles in autoimmune diseases might result from combinations of these ligands and their corresponding killer cell immunoglobulin-like receptors (KIR) that were expressed by natural killer (NK) cells and a subset of T-lymphocytes.[12], [13] Moreover, the importance of HLA-DP alleles was highlighted in genome-wide association studies (GWAS) and comprehensive HLA analyses of patients with autoimmune diseases, which demonstrated HLA-DP gene variations having a strong association with systemic lupus erythematosus, antineutrophil cytoplasmic antibody-associated vasculitis, and granulomatosis with polyangiitis.[14]–[16] Based on the above reports, we searched for associations of particular HLA alleles, including HLA class I (A, B, and C) and HLA class II (DRB1, DQB1, and DPB1), and haplotypes with susceptibility, clinical manifestations, and outcome of patients with AIH.

## Materials and Methods

### Ethics statement

This study was approved by the ethical committee of Shinshu University School of Medicine, Matsumoto, Japan, and written informed consent was obtained from all subjects. The study was conducted in accordance with the principles of the Declaration of Helsinki.

### Subjects

Between January 1979 and March 2013, 156 patients of Japanese descent with type 1 AIH were enrolled in this study. Their clinical and laboratory data at the time of diagnosis are shown in Table 1. The median follow-up period was 118 months (range: 6–403 months). The HLA class I and II allelic genotypes of 201 healthy subjects that were obtained in a previous study [17] were adopted as controls. Normal subjects were unrelated healthy apheresis blood donors living in the central region of Japan.[17] All cases of AIH had been diagnosed according to the scoring system from the International Autoimmune Hepatitis Group.[18] All subjects were negative for the hepatitis B surface antigen, antibody to hepatitis B core antigen, and antibody to hepatitis C in serum samples and exhibited no evidence of other liver diseases. Alanine aminotransferase (ALT), aspartate aminotransferase (AST), and other relevant biochemical tests were performed using standard methods.[19] Anti-nuclear antibody (ANA) and anti-smooth muscle antibody (SMA) were determined as reported previously.[20] Liver cirrhosis was diagnosed by histological examination and/or characteristic clinical signs of advanced liver disease.[21] HCC was diagnosed by histological examination and/or imaging studies, and hepatic failure was diagnosed by the presence of esophageal varices, ascites, and hepatic encephalopathy. During the follow-up, cirrhosis, hepatic failure, and HCC developed in 16% (25/156), 6% (9/156), and 4% (6/156) of patients.

**Table 1:** Demographic and Clinical Characteristics of 156 Patients with Type 1 AIH.

Clinical feature		
Age at diagnosis (years)	62	(57–66)
Observation period (months)	118	(6–403)
Female, n (%)	138	(89)
AIH score	16	(10–23)
Albumin (4.2–5.1 g/dL)	3.7	(1.7–4.6)
AST (12–37 IU/L)	421	(30–5586)
ALT (7–45 IU/L)	494	(21–7436)
Bilirubin (0.3–1.2 mg/dL)	1.9	(0.4–30.2)
IgG (870–1700 mg/dL)	2770	(826–7248)
ANA (<×40), n (%)	150	(96)
SMA (<×40), n (%)	66/112	(59)

Values are expressed as median (range) unless otherwise noted.

Abbreviations: ALT, alanine aminotransferase; AST, aspartate aminotransferase; ANA, anti-nuclear antibody; SMA, anti-smooth muscle antibody.

### HLA Class I and II Typing

Genomic DNA from patients and healthy individuals was isolated by phenolic extraction of sodium dodecyl sulfate-lysed and proteinase K-treated cells, as described previously.[22] HLA typing was carried out using a Luminex multi-analyzer profiling system with a LAB type SSO One Lambda typing kit (One Lambda, Inc. Canoga Park, CA). HLA genotypes were determined by sequence-based typing, as earlier described.[23] HLA-Bw4, HLA-Bw6, HLA-C1, and HLA-C2 KIR ligands were assigned based on the amino acid residues of the HLA-A, HLA-B, and HLA-C alleles. The peptide sequences of all HLA-DRB1, DQB1, and DPB1 alleles in the IMGT/HLA database release 3.14.0 (October 2013) were aligned.

### Statistical Analysis

Phenotype frequencies were estimated by direct counting for each HLA allele. The significance of an association was evaluated using chi-square analysis or Fisher's exact test. *P* values were subjected to Bonferroni correction by multiplication by the number of different alleles observed in each locus (*Pc*). The Mann-Whitney U test was used to analyze continuous variables where appropriate. A *P* value of less than 0.05 was considered to be statistically significant. Association strength was estimated by calculating the odds ratio (OR) and 95% confidence interval (CI).

## Results

### Associations of HLA Alleles

HLA class I (A, B, and C) and class II (DRB1, DQB1, DPB1) were genotyped in 156 patients with AIH. Among the HLA class I alleles, the frequency of A*24:02 and C*01:02 were significantly increased in patients with AIH compared with healthy subjects (35% vs. 22%, *Pc* = 0.0053; OR = 1.84, 95% CI = 1.32–2.55, and 23% vs. 14%, *Pc* = 0.036; OR = 1.81, 95% CI = 1.24–2.65, respectively) (Table 2). On the other hand, the frequency of the HLA-C2 allele was significantly reduced in AIH as compared with healthy controls (6% vs. 13%, *P* = 0.0054; OR = 2.12, 95% CI = 1.24–3.64). Patients who were homozygous for the HLA-C1 allele (without the protective HLA-C2 variant) exhibited an increased risk of AIH (88% vs. 76%, *P* = 0.0036; OR = 2.32, 95% CI = 1.30–4.14). The frequency of the HLA-Bw4 allele was comparable between patients and controls (27% vs. 26%).

**Table 2:** Statistical Analysis of Representative HLA Alleles among Patients with Type 1 AIH and Healthy Subjects.

Allele	Frequency, n (%)		*P* Value	*Pc* Value	OR (95% CI)
	Patients (2n = 312)	Controls (2n = 402)			
A*02:01	34 (11)	65 (16)	0.043	>0.1	0.63 (0.41–0.99)
A*24:02	108 (35)	90 (22)	0.00029	**0.0053**	1.84 (1.32–2.55)
A*31:01	36 (12)	28 (7)	0.034	>0.1	1.74 (1.04–2.92)
B*15:01	19 (6)	50 (12)	0.0044	>0.1	0.46 (0.26–0.79)
B*39:01	11 (4)	28 (7)	0.045	>0.1	0.49 (0.24–1.00)
B*44:03	18 (6)	41 (10)	0.033	>0.1	0.54 (0.30–0.96)
B*52:01	32 (10)	22 (6)	0.016	>0.1	1.97 (1.12–3.47)
B*54:01	40 (13)	29 (7)	0.012	>0.1	1.89 (1.14–3.13)
C*01:02	73 (23)	58 (14)	0.0021	**0.036**	1.81 (1.24–2.65)
C*07:02	34 (11)	69 (17)	0.018	>0.1	0.59 (0.38–0.92)
C*12:02	30 (10)	22 (6)	0.035	>0.1	1.84 (1.04–3.25)
C*14:03	17 (5)	40 (10)	0.028	>0.1	0.52 (0.29–0.94)
DRB1*01:01	8 (3)	24 (6)	0.029	>0.1	0.41 (0.18–0.94)
DRB1*04:03	4 (1)	18 (5)	0.026	>0.1	0.28 (0.09–0.83)
DRB1*04:05	95 (30)	45 (11)	1.3×10^−10^	**4.0×10^−9^**	3.47 (2.34–5.14)
DRB1*04:06	5 (2)	18 (5)	0.031	>0.1	0.35 (0.13–0.95)
DRB1*13:02	13 (4)	32 (8)	0.039	>0.1	0.50 (0.26–0.98)
DRB1*14:54	6 (2)	20 (5)	0.031	>0.1	0.37 (0.15–0.94)
DRB1*15:01	20 (6)	54 (13)	0.0023	0.068	0.44 (0.26–0.75)
DQB1*03:02	19 (6)	48 (12)	0.0078	>0.1	0.48 (0.28–0.83)
DQB1*04:01	94 (30)	45 (11)	2.3×10^−10^	**3.7×10^−9^**	3.42 (2.31–5.07)
DQB1*05:01	9 (3)	31 (8)	0.0054	0.087	0.36 (0.17–0.76)
DQB1*06:02	17 (5)	53 (13)	0.0006	**0.009**	0.38 (0.22–0.67)
DPB1*04:02	23 (7)	52 (13)	0.016	>0.1	0.54 (0.32–0.90)
DPB1*05:01	143 (46)	152 (38)	0.031	>0.1	1.39 (1.03–1.88)

Abbreviations: Pc, corrected P; OR, odds ratio; CI, confidence interval.

Among the HLA class II alleles, DRB1*04:05 and DQB1*04:01 were significantly associated with AIH compared with healthy subjects (30% vs. 11%, *Pc* = 4.0×10^−9^; OR = 3.47, 95% CI = 2.34–5.14, and 30% vs. 11%, *Pc* = 3.7×10^−9^; OR = 3.42, 95% CI = 2.31–5.07, respectively), as reported previously (Table 2). Conversely, DRB1*15:01 (6% vs.13%; *Pc* = 0.068) and DQB1*06:02 (5% vs. 13%; *Pc* = 0.009; OR = 0.38, 95% CI = 0.22–0.67) conferred a reduced risk of AIH occurrence. However, reevaluation of these alleles after excluding DRB1*04:05 and DQB1*04:01 carriers from the analysis resulted in no significant differences in the frequencies of DRB1*15:01 (9% vs. 15%; *Pc*>0.1) or DQB1*06:02 (9% vs. 15%; *Pc*>0.1). The DPB1*05:01 allele was found at an increased frequency in patients with AIH, which suggested an effect on susceptibility (46% vs. 38%), but this difference was not significant after correction for multiple testing.

### Associations of HLA Haplotypes

The frequency of the DRB1*04:05-DQB1*04:01 haplotype in patients with AIH was 30% and significantly higher than the 11% observed in healthy subjects (*P* = 1.2×10^−10^; OR = 3.51, 95% CI = 2.36–5.21) (Table 3). The DRB1*04:05-DQB1*04:01-DPB1*05:01 haplotype was also significantly correlated with disease (22% vs. 7%, *P* = 4.6×10^−9^; OR = 3.79, 95% CI = 2.38–6.06). The A*24:02 and C*01:02 alleles, which were implicated in AIH susceptibility, were included in the fourth most frequent haplotype (A*24:02-C*01:02-B*54:01-DRB1*04:05-DQB1*04:01) in our cohort. Whereas the DRB1*04:05-DQB1*04:01 haplotype showed the strongest association with disease onset, protective effects were observed for DRB1*15:01-DQB1*06:02 (5% vs. 13%, *P* = 0.00057; OR = 0.38, 95% CI = 0.22–0.67).

**Table 3:** Number of Individuals with Haplotypes Containing HLA-DRB1*04:05-DQB1*04:01.

Haplotype	Patients (2n = 312)	Controls (2n = 402)	*P* value
A*x -C*x- B*x- DRB1*04:05-DQB1*04:01-DPB1*x	94 (30%)	44 (11%)	**1.2×10^−10^**
A*x -C*x- B*x- DRB1*04:05-DQB1*04:01-DPB1*05:01	69 (22%)	28 (7%)	**4.6×10^−9^**
A*x- C*01:02-B*x- DRB1*04:05-DQB1*04:01-DPB1*x	55 (18%)	26 (6%)	**3.1×10^−6^**
A*x- C*01:02-B*54:01-DRB1*04:05-DQB1*04:01-DPB1*x	36 (12%)	13 (3%)	**1.3×10^−5^**
A*24:02-C*x -B*x- DRB1*04:05-DQB1*04:01-DPB1*x	44 (14%)	22 (5%)	**7.8×10^−5^**
A*24:02-C*01:02-B*x- DRB1*04:05-DQB1*04:01-DPB1*x	28 (9%)	14 (3%)	**0.0020**
A*24:02-C*01:02-B*54:01-DRB1*04:05-DQB1*04:01-DPB1*05:01	19 (6%)	8 (2%)	**0.0044**

x represents any allele at that locus, including A*24:02, C*01:02, B*54:01, and DPB1*05:01.

### Associations between HLA and Clinical Findings

According to clinical and laboratory data, median serum IgG was significantly higher in patients with the DRB1*04:05-DQB1*04:01 haplotype than in those without (3042 vs. 2606 mg/dL, *P* = 0.041). This was also the case for SMA positivity (50 of 65 [77%] vs. 16 of 47 [34%], *P* = 0.000006; OR = 6.46). There were no significant differences in the serum levels of albumin, ALT, AST, or bilirubin, nor was there in the frequency of elevated ANA, between patients with or without DRB1*04:05.

We next stratified AIH patients according to the development of HCC and hepatic failure. The HLA-DRB1*15:01 and DQB1*06:02 alleles (25% vs. 6%, *P* = 0.038; OR = 5.55, 95% CI = 1.37–22.40, and 25% vs. 5%, *P* = 0.017; OR = 6.81, 95% CI = 1.66–27.96, respectively) and the DRB1*15:01-DQB1*06:02 haplotype (25% vs. 5%, *P* = 0.017; OR = 6.81, 95% CI = 1.66–27.96) were all found to be significantly associated with the development of HCC. When AIH patients with hepatic failure were compared with those without, significant genetic associations of the DRB1*08:03 allele and DRB1*08:03-DQB1*06:01 haplotype (22% vs. 6%, *P* = 0.034; OR = 4.38, 95% CI = 1.31–14.68) were seen. No other haplotypes were associated with cirrhosis.

### Amino Acid Residues in HLA-DRB1, DQB1, and DPB1

**Figure 1:**
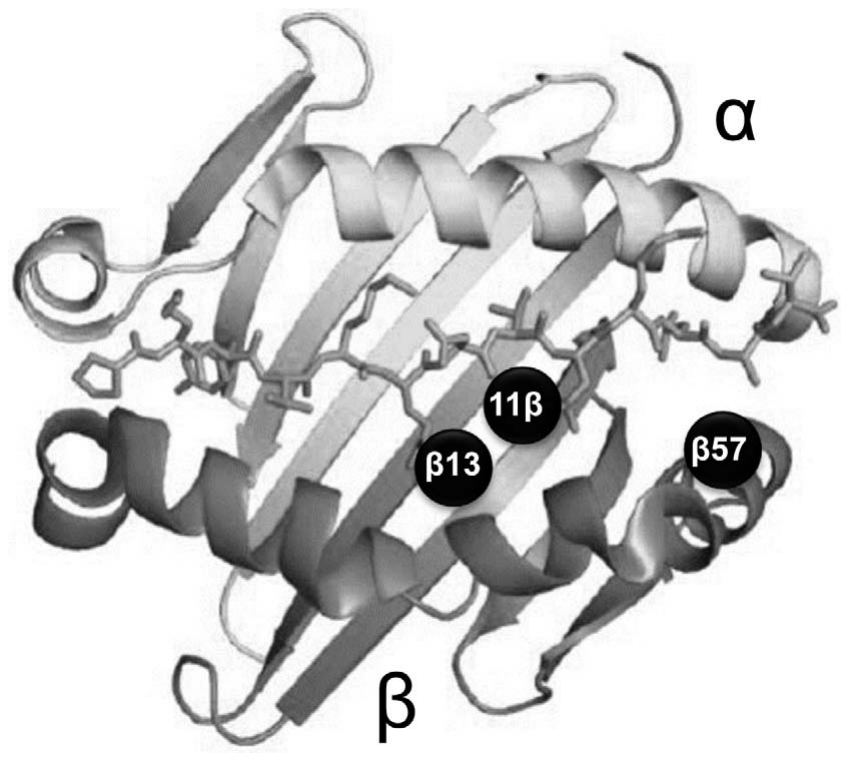
Relative amino acid positions at β11, β13, and β57 on the HLA-DRB1 molecule. Three-dimensional structure of HLA-DRB1 adapted from Stern et al. [40] The molecule is composed of 2 opposing α-helices and a series of supporting β-pleated sheets. The relative positions of the 3 amino acids discussed in this study are indicated by black spots.

The amino acid sequences encoded by the second exon of *HLA-DRB1, DQB1,* and *DPB1* were determined for each subject. As shown in Table 4, the prevalence of valine at position 11 (*P* = 1.4×10^−6^; OR = 2.19), histidine at position 13 (*P* = 1.3×10^−7^; OR = 2.38), and serine at position 57 (*P* = 2.3×10^−8^; OR = 2.53) in DRβ1 was significantly higher in patients with AIH compared with healthy subjects. The amino acid residue at position 13 affects the binding of antigen side chains associated with the fourth and sixth pockets of the expressed DR molecule, while the amino acid residues at positions 11 and 57 influence the binding of antigen side chains associated with the sixth and ninth binding pockets, respectively (Figure 1). The amino acids at positions 11, 13, and 57 in HLA DRβ1 consisted of 12 haplotypes (Table 4). Valine-histidine-serine residues conferred a significantly elevated risk of AIH (*P* = 1.7×10^−11^; OR = 3.52), whereas serine-serine-aspartic acid, leucine-phenylalanine-aspartic acid, and serine-serine-alanine apparently imparted protection against the disease (*P* = 0.035; OR = 0.62, *P* = 0.029; OR = 0.41, and *P* = 0.042; OR = 0.42, respectively). Amino acids in DQβ1 that were associated with disease susceptibility included glycine at position 26 (*P* = 1.8×10^−5^; OR = 1.97) and leucine at positions 53 (*P* = 9.0×10^−6^; OR = 1.99) and 56 (*P* = 2.2×10^−11^; OR = 3.36). There were no significant associations among DPβ1 amino acids.

**Table 4:** Influence of Three Amino Acid Positions in HLA-DRβ1 Associated with AIH Risk or Protection.

Classic HLA-DRB1 allele	HLA-DRβ1 amino acid at position			Allele frequency		*P* value	OR	95% CI
	11	13	57	Patients	Controls			
*04:05, *04:10, *04:17	Val	His	Ser	0.333	0.124	**1.7×10^−11^**	3.52	2.41–5.14
*15:01, *15:02, *16:02	Pro	Arg	Asp	0.160	0.199	0.18	0.77	0.52–1.13
*09:01	Asp	Phe	Val	0.112	0.102	0.66	1.11	0.69–1.79
*11:01, *13:01, *13:02, *14:03, *14:05, *14:06, *14;18	Ser	Ser	Asp	0.109	0.164	**0.035**	0.62	0.40–097
*04:01, *04:03, *04:04, *04:06, *04:07, *04:59	Val	His	Asp	0.077	0.102	0.25	0.73	0.43–1.24
*08:03	Ser	Gly	Ser	0.071	0.087	0.42	0.8	0.46–1.39
*12:01, *12:02	Ser	Gly	Val	0.048	0.052	0.80	0.92	0.46–1.81
*08:02, *08:09	Ser	Gly	Asp	0.035	0.037	0.88	0.94	0.43–2.08
*01:01	Leu	Phe	Asp	0.026	0.060	**0.029**	0.41	0.18–0.94
*14:07, *14:54	Ser	Ser	Ala	0.022	0.052	**0.042**	0.42	0.08–0.99
*07:01	Gly	Tyr	Val	0.003	0.002	0.59	1.29	0.08–20.7
*10:01	Val	Phe	Asp	0.003	0.017	0.15	0.18	0.02–1.48

Abbreviations: HLA, human leukocyte antigen; AIH, autoimmune hepatitis; OR, odds ratio; CI, confidence interval.

## Discussion

The present study of a larger new cohort of Japanese patients with AIH confirmed that the HLA-DRB1*04:05 (*Pc* = 3.9×10^−9^) and DQB1*04:01 (*Pc* = 3.7×10^−9^) alleles and the DRB1*04:05-DQB1*04:01 haplotype (*Pc* = 2.3×10^−10^) are the principal susceptibility alleles for type 1 AIH. As DRB1*04:05 is known to be in linkage disequilibrium with DQB1*04:01 in the Japanese population, either allele may presumably be associated with susceptibility to AIH. However, because the relative linkage disequilibrium value for both alleles is 100% in Japan, we cannot presently elucidate exactly which allele is associated disease susceptibility. In general, *HLA-DP* genes have been somewhat neglected in terms of their impact on human disease relative to *HLA-DR* and -*DQ*, partly because *HLA-DPA1* and -*DPB1* are less polymorphic and also due to the fact that HLA-DP cell surface expression levels tend to be lower than those of HLA-DR and -DQ. Since accumulating data had indicated that *HLA-DP* alleles were associated with various autoimmune diseases,[16], [24]–[27] we investigated whether they influenced susceptibility to AIH but found no significant associations.

Prior studies have proposed a histidine residue at position 13 of the DRβ-polypeptide to be a critical determinant of disease susceptibility in Japan,[28] in contrast to a lysine residue at position 71 of the DRβ-polypeptide in patients of European descent.[6], [7] In the present study, the incidence of valine-11 (OR = 2.19), histidine-13 (OR = 2.38), and serine-57 (OR = 2.53) encoded by DRB1*04:05 was significantly higher in AIH patients. Moreover, a specific haplotype determined by the amino acids valine-histidine-serine at positions 11, 13, and 57 in DRβ1 was strongly associated with AIH. This finding is punctuated by the central location of these residues in the peptide-binding groove of HLA-DRβ1 in AIH etiology. Positions 11 and 13 are located in the β-sheet floor with their side chains oriented toward the peptide-binding groove. Meanwhile, the amino acid residue at position 57 influences the binding of antigen side chains associated with the ninth pocket of the expressed DR molecule, which might factor predominantly in susceptibility to AIH in the Japanese. Especially because the *HLA*-DRB1*04:05 allele is 95% comprised of the haplotype valine-histidine-arginine-alanine in Japan, this allele can be said to play a critical role in AIH pathogenesis. A single DPB1 amino acid, glutamic acid at position 69, has been shown to contribute to graft-versus-host disease in otherwise identical HLA sibling bone marrow transplantation[29] and factor in the susceptibility to Beryllium disease.[24], [26] However, the frequency of glutamic acid at position 69 in our patients with AIH and controls was 35% and 39%, respectively. Hence, amino acid residues in DPβ1 were not implicated in disease susceptibility.

Although our prior report showed that no HLA class I alleles were involved with susceptibility to AIH,[4] this considerably larger study of new patients uncovered that the A*24:02 (*Pc* = 0.0053) and C*01:02 (*Pc* = 0.036) alleles were associated with type 1 AIH in the Japanese population. However, neither of these is believed to be a primary susceptibility allele in AIH. The most likely explanation for our observations is that these alleles reflect the known linkage disequilibrium of the HLA-A*24:02-C*01:02-DRB1*04:05-DQB1*04:01 haplotype in the Japanese. This interpretation is supported by the observation that the OR of AIH with DQB1*04:01 is greater than those with A*24:02, and/or C*01:02 (Table 3).

To our knowledge, this is the first study revealing that the DRB1*15:01-DQB1*06:02 haplotype may play a protective role against AIH in the Japanese. Our data support the finding that the DRB1*15:01 allele and/or DRB1*15:01-DQB1*06:02 haplotype has a significantly reduced incidence and apparently protective role in Caucasian patients with AIH.[7], [30]_ENREF_26 This is a newly established consensus on this haplotype conferring AIH resistance. However, the protective DRB1*15:01-DQB1*06:02 haplotype may not be common to autoimmune liver diseases since the DRB1*11 and *13 alleles are resistant to PBC development in Caucasians and Japanese;[23], [31], [32] we cannot exclude the possibility that these associations are simply linkage markers for a yet undefined gene in AIH. Moreover, this protective role may have occurred as a consequence of the increased frequency of the DRB1*04:05-DQB1*04:01 haplotype since a significant difference was lost after excluding DRB1*04:05 and DQB1*04:01 carriers. Our finding may have also been influenced by the small sample size of this study.

Our results uncovered significant associations between the DRB1*04:05-DQB1*04:01 haplotype and elevated serum IgG and SMA positivity. Since the autoantibodies involved in type 1 AIH are neither pathogenic nor organ-specific, they are more useful as diagnostic tools than as instruments of pathogenesis.[8] Such associations between HLA alleles or haplotypes and IgG levels have not been reported elsewhere, and the relationship of this particular HLA haplotype with the presence of autoantibodies and IgG is intriguing.

With regard to disease progression, this study revealed novel associations of the DRB1*15:01-DQB1*06:02 haplotype with the development of HCC and the DRB1*08:03-DQB1*06:01 haplotype with hepatic failure. Recent retrospective [10], [33]–[36] and population-based [37], [38] studies have shown that HCC complicating AIH is not as rare as earlier believed. Although these reports suggested that male gender, cirrhosis at presentation, elderly age, and/or abnormal ALT were risk factors in the development of HCC, few studies assessed the relationship between HCC and HLA alleles. Montaro-Loza et al.[33] and our group [10] evaluated the association of several HLA alleles, including DRB1*04 and/or DRB1*03, with the development of HCC and discovered no statistical associations. Moreover, the genetic risk factors contributing to hepatic failure have not been precisely assessed in AIH. Hence, although the reasons underlying these observations are unknown, the association between HLA haplotypes and disease progression is striking. Interpretations of our findings need to be tempered because the study was retrospective in nature and of a small sample size despite its long median follow-up period of 118 months. As only 6 (4%) and 9 (6%) of our 156 patients experienced HCC and hepatic failure, respectively, future prospective follow-up studies in larger cohorts are required. Furthermore, GWAS are warranted to detect other genes influencing the susceptibility, pathogenesis, and progression of AIH.

In conclusion, the DRB1*04:05-DQB1*04:01 and DRB1*15:01-DQB1*06:02 haplotypes are associated with AIH susceptibility and protection, respectively, in the Japanese population. DRB1*04:05-DQB1*04:01 is associated with elevated serum IgG and SMA positivity. DRB1*15:01-DQB1*06:02 as well as DRB1*08:03-DQB1*06:01 are novel haplotypes that are related to AIH progression. In addition, specific amino acid residues in the DRβchain appear to contribute to susceptibility or resistance to AIH. We have recently developed super high-resolution single-molecule sequenced-based typing of HLA loci using next generation sequencing.[39] This method is able to amplify entire HLA gene sequences from the enhancer-promoter region to the 3′ untranslated region and detect 8-digit level HLA alleles. With this technique, resequencing of the entire HLA region is expected to provide more precise genetic information on susceptibility and progression in AIH in Japan.
