# Correction: Human Leukocyte Antigen Class II Haplotypes Affect Clinical Characteristics and Progression of Type 1 Autoimmune Hepatitis in Japan

**DOI:** 10.1371/journal.pone.0109494

**Published:** 2014-09-26

**Authors:** The PLOS ONE Staff

The second sentence of the abstract incorrectly cites the number of healthy patients as 210. The correct number of healthy patients is 201, and the correct sentence is: “We reinvestigated HLA class I *A*, *B*, and *C* and HLA class II *DRB1*, *DQB1*, and *DPB1* alleles and haplotypes in a larger new cohort of 156 Japanese patients with type 1 AIH and compared them with the published data of 201 healthy subjects.”

The third sentence of the abstract incorrectly refers to “anti-smooth antigen positivity.” The correct phrase is “anti-smooth antibody positivity,” and the correct sentence is “The DRB1*04:05-DQB1*04:01 haplotype was significantly associated with AIH susceptibility (30% vs. 11%, *P*  =  1.2×10^−10^; odds ratio [OR]  =  3.51) and correlated with elevated serum IgG (3042 vs. 2606 mg/dL, *P*  =  0.041) and anti-smooth muscle antibody positivity (77% vs. 34%, *P*  =  0.000006).”

## References

[1] Umemura T, Katsuyama Y, Yoshizawa K, Kimura T, Joshita S, et al (2014) Human Leukocyte Antigen Class II Haplotypes Affect Clinical Characteristics and Progression of Type 1 Autoimmune Hepatitis in Japan. PLoS ONE 9(6): e100565 doi:10.1371/journal.pone.0100565 24956105 10.1371/journal.pone.0100565 PMC4067340

